# TLR2 Expression on Select Lymphocyte Subsets as a New Marker in Glomerulonephritis

**DOI:** 10.3390/jcm9020541

**Published:** 2020-02-17

**Authors:** Sebastian Mertowski, Ewelina Grywalska, Krzysztof Gosik, Iwona Smarz-Widelska, Anna Hymos, Grzegorz Dworacki, Paulina Niedźwiedzka-Rystwej, Bartłomiej Drop, Jacek Roliński, Wojciech Załuska

**Affiliations:** 1 Department of Clinical Immunology and Immunotherapy, Medical University of Lublin, Lublin 20-093, Poland; krzysiekgoja@gmail.com (K.G.); jacek.rolinski@gmail.com (J.R.); 2 Department of Immunology, St. John’s Cancer Centre, Lublin 20-090, Poland; 3 Department of Nephrology, Cardinal Stefan Wyszynski Provincial Hospital in Lublin, Lublin 20-718, Poland; i.widelska@interia.pl; 4 Department of Otolaryngology and Laryngeal Oncology, Medical University of Lublin, Lublin 20-954, Poland; annahymos@gmail.com; 5 Department of Immunology, Poznan University of Medical Sciences, Poznan 60-806, Poland; gdwrck@ump.edu.pl; 6 Institute of Biology, University of Szczecin, Szczecin 71-412, Poland; paulina.niedzwiedzka@gmail.com; 7 Department of Informatics and Medical Statistics, Medical University of Lublin, Lublin 20-090, Poland; bartlomiej.drop@umlub.pl; 8 Department of Nephrology, Medical University of Lublin, Lublin 20-954, Poland; wojciech.zaluska@umlub.pl

**Keywords:** TLR2, glomerulonephritis, immunology, CD4+ T lymphocyte, CD8+ T lymphocyte, CD19+ B lymphocyte

## Abstract

Toll-like receptor (TLR) signaling may be involved in autoimmune kidney disorders and has been implicated in proliferative and non-proliferative glomerulonephritis (PGN and NPGN). In this study, we investigated the expression of TLR2 on T and B lymphocytes in relation to selected clinical parameters in patients with PGN and NPGN. We collected peripheral blood from the ulnar vein of patients with PGN (*n* = 15) or NPGN (*n* = 22) and healthy volunteers (*n* = 20). The percentage of peripheral blood mononuclear cells expressing TLR2 was determined with flow cytometry. TLR2 expression on T and B lymphocytes was increased in PGN patients compared with NPGN patients and controls (*p* ≤ 0.001). In patients with PGN, TLR2 expression correlated negatively with the serum concentrations of IgG and albumin and positively with urine protein excretion. Receiver operating characteristic (ROC) analysis indicated that TLR2 expression is a highly specific marker to distinguish PGN patients from NPGN patients and controls, especially on CD4+ T lymphocytes. Its use as a non-invasive marker of disease should be further investigated.

## 1. Introduction

Glomerulonephritis is an umbrella term that encompasses several diseases involving the inflammation of the glomeruli, the units of filtration of the kidneys. Proliferative glomerulonephritis (PGN) is characterized by an increased number of cells in the glomeruli, while in non-proliferative glomerulonephritis (NPGN) the number of cells does not change. In these diseases, inflammation of the cells that surround the glomeruli leads to an increased permeability to proteins, which are then excreted in the urine (proteinuria) [1]. Patients usually develop nephrotic or nephritic syndromes, or both [2]. In addition to proteinuria, nephrotic syndrome is characterized by hypoalbuminemia and hyperlipidemia [3,4], while nephritic syndrome is characterized by blood in the urine and decreased urine production [5,6]. Importantly, the disease course is markedly different in PGN and NPGN. NPGN is typically associated with nephrotic syndrome and a good prognosis, whereas PGN causes nephritic syndrome and can rapidly lead to end-stage renal failure. It is crucial to differentiate between these two types of glomerulonephritis to choose an appropriate treatment. However, the distinction between PGN and NPGN requires a kidney biopsy because non-invasive markers are lacking.

Toll-like receptors (TLRs) are part of the pattern recognition receptors (PRRs) of the innate immune system. They recognize pathogen-associated molecular patterns (PAMPs) such as the components of the bacterial cell wall. Studies have shown that TLRs can also be activated by endogenous molecules associated with damage to the body’s own cells, or danger-associated molecular patterns (DAMPs) [7]. When TLRs are activated, the transcription factor NF-κB is released, and this leads to the production of inflammatory mediators such as IL-1, IL-2, IL-6, IL-12, and TNF-α [8,9]. TLRs are also involved in the activation of the adaptive immune system by antigen-presenting cells that promote CD4 helper cell differentiation, B cell activation, and antibody production.

TLR2 is a membrane-bound receptor found on cells of the immune system and on non-immune cells, like those in the renal tubules and Bowman’s capsule [10]. It recognizes molecules of microbial origin, fungal zymosan, and viral glycoprotein and hemagglutinin. TLR2 also has endogenous ligands, such as the intracellular protein HSP70 [11,12,13,14]. 

Due to its role in the immune response, TLR signaling may play a part in autoimmune disorders affecting the kidneys. TLRs have been implicated in systemic lupus erythematosus [15,16] and immunoglobulin (Ig) A nephropathy [17]. Furthermore, many of the kidney diseases are the consequences of various infections. As such, a pathogen-induced TLR activation has been implicated in glomerulonephritis and lipopeptide, a TLR2 agonist expressed by bacteria has been shown to intensify glomerulonephritis by activating TLR2 [18,19,20].

Non-invasive methods to distinguish PGN from NPG are lacking. In this study, we investigated whether TLR2 expression in immune cells could differentiate between PGN and NPGN. 

## 2. Results

### 2.1. Characteristics of the Patients and Controls 

The mean age of the patients was 41.77 years in the NPGN group and 46 years in the PGN group. The characteristics of the patients and controls, including the levels of selected proteins, complement components, and parameters related to renal function, are presented in Table 1. The complete blood count and the concentration of the basic lymphocyte subsets of the patients and controls are presented in Table 2. The frequency of TLR2 on T and B cells is shown in Table 3.

### 2.2. Comparisons of Protein Concentrations, Complement Components, Renal Function Parameters, and Blood Cell Counts between Patients with PGN, NPGN, and Controls

The patients with NPGN had higher urea and blood urea nitrogen (BUN) concentrations than PGN patients and controls, but only the difference with the controls was significant (*p* = 0.034). The urea concentration in the NPGN group was 20% higher than the normal range for healthy people, and the BUN concentration was nearly 37% over the normal range. Urea and BUN concentrations in the PGN group were also higher than in the control group, but the differences were not statistically significant (Table 1). Both groups of patients had a significantly lower IgG concentration compared to control, with the concentration in NPGN patients being lower than in PGN patients (*p* = 0.068). NPGN patients had an IgA concentration that was statistically lower than that of PGN patients (*p* = 0.025), but it was not significantly different from that of the controls (*p* = 0.431)). Regarding the total concentration of protein in serum, and the concentration of albumin, both groups of patients had a significantly lower value than the control group (*p* < 0.001). Both groups of patients had a significantly higher quantity of protein after a 24-h urine collection test compared to control, with the concentration in the NPGN group being higher than in the PGN group (*p* = 0.072; Table 1). Patients in the PGN group had a significantly higher hemoglobin concentration compared to the control group (*p* = 0.022; Table 2).

### 2.3. Comparisons of TLR2 Expression on T and B Lymphocyte Subsets between Patients with PGN, NPGN, and Controls

The percentages of CD4+ T cells, CD8+ T cells, and CD19+ B cells expressing TLR2 in the PGN group were significantly higher than those in the NPGN group and the control group (*p* ≤ 0.001; Table 3). Compared to the control group, the percentages of these lymphocyte subsets expressing TLR2 also appeared higher in the NPGN group, but only the difference in the frequency of CD4+TLR-2+ cells was significant (*p* = 0.003, Table 3).

### 2.4. Correlations between TLR2 Expression on T and B Lymphocyte Subsets and Selected Laboratory Parameters in NPGN and PGN Patients

**Figure 1:**
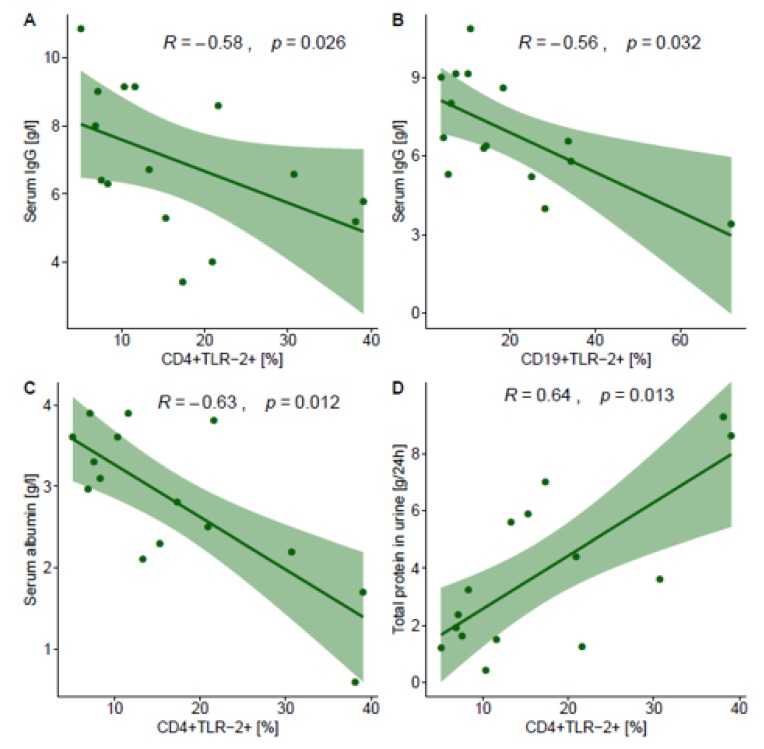
Correlation between the expression of TLR-2 and selected laboratory measures in patients with PGN. R: Spearman correlation coefficient. (**A**). Correlation between the frequencies of CD4+TLR-2+ cells and the concentration of IgG (rho = −0.58, *p* = 0.026). (**B**). Correlation between the frequencies of CD19+TLR-2+ cells and the concentration of IgG (rho = −0.56, *p* = 0.032). (**C**). Correlation between the frequencies of CD4+TLR-2+ cells and the concentration of albumin (rho = −0.63, *p* = 0.012). (**D**). Correlation between the frequencies of CD4+TLR-2+ cells and the urinary protein excretion (rho = 0.64, *p* = 0.013).

Next, we investigated the relationship between TLR2 expression and the laboratory parameters that showed statistical differences (i.e., urine protein excretion and the serum concentrations of urea, BUN, IgG, IgA, total protein, and albumin). In patients with PGN, the frequencies of CD4+TLR-2+ cells and CD19+TLR-2+ cells correlated negatively with the concentration of IgG (rho = −0.579, *p* = 0.026; rho = −0.561, *p* = 0.032, respectively; Figure 1). Moreover, the frequency of CD4+TLR-2+ cells in patients with PGN correlated negatively with the concentration of albumin (rho = −0.631, p=0.012; Figure 1) and positively with urinary protein excretion (rho = 0.636, *p* = 0.013). The remaining correlations were not significant. Table 4 shows all Spearman correlation coefficients. 

### 2.5. Receiver Operating Characteristic (ROC) Curve Analysis to Determine The Diagnostic Accuracy of TLR2 Expression on T and B Lymphocytes in Patients with PGN vs NPGN and in Patients vs Controls

**Figure 2:**
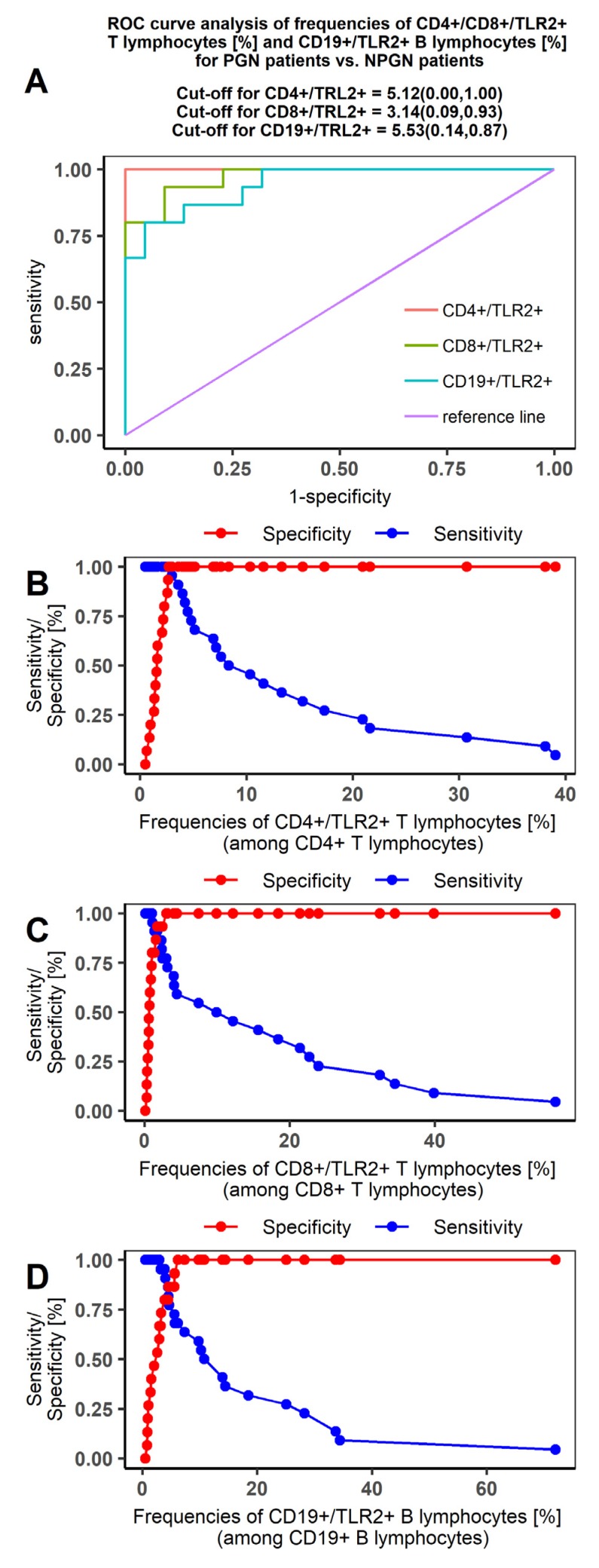
Receiver operating characteristic (ROC) curve analysis showing the sensitivity and specificity of TLR2 expression to distinguish between patients with non-proliferative (NPGN) and proliferative glomerulonephritis (PGN). (**A**) ROC curve analysis for CD4+/CD8+/TLR2+ T lymphocyte (%) and CD19+/TLR2+ B lymphocyte frequencies (%); (**B**) sensitivity/specificity curve for CD4+/TLR2+T lymphocytes (%); (**C**) sensitivity/specificity curve for CD8+/TLR2+ T lymphocytes (%); (**D**) sensitivity/specificity curve for CD19+/TLR2+B lymphocytes.

Our ROC curves comparing PGN and NPGN patients were characterized by high sensitivity for each of the T and B lymphocyte datasets (Figure 2). The area under the curve (AUC) value indicated high specificity for distinguishing PGN from NPGN patients. Furthermore, the most differentiating indicator for both groups of patients was the expression of TLR2 on the CD4+ T lymphocyte subset (AUC = 1; Table 5). 

**Figure 3:**
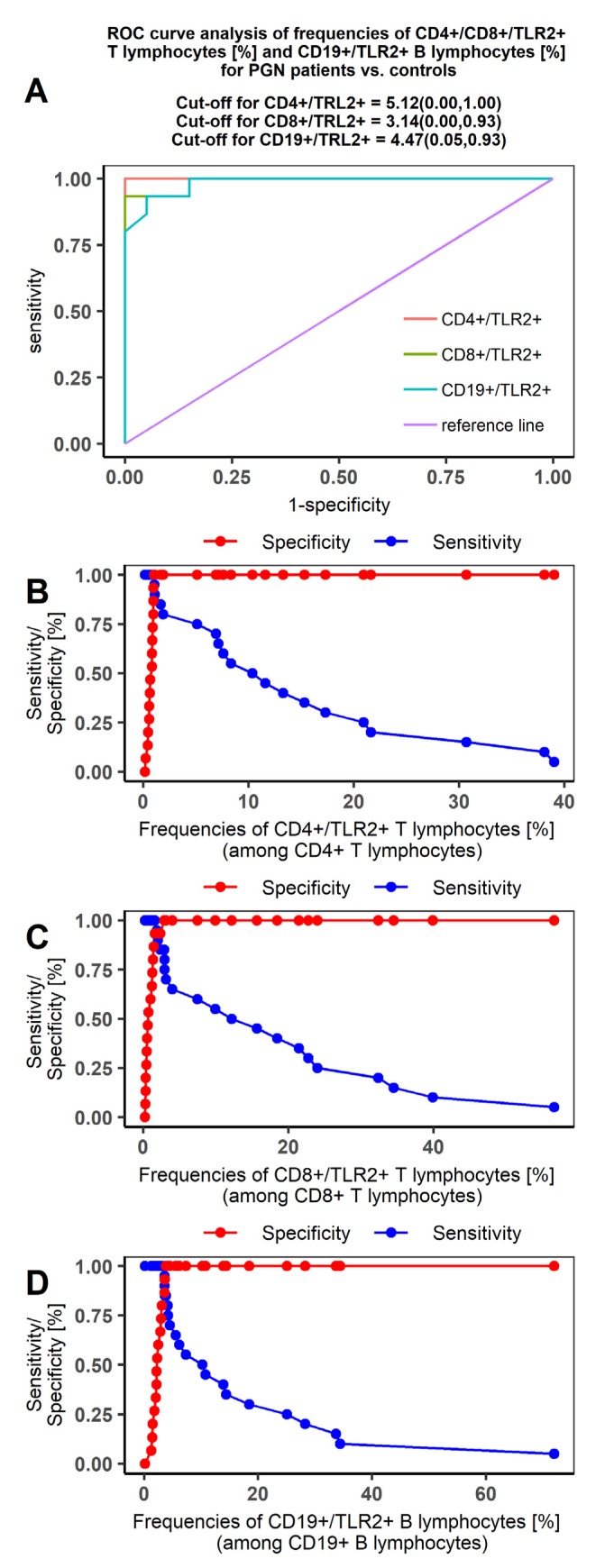
Receiver operating characteristic (ROC) curve analysis on the sensitivity and specificity of TLR2 expression to distinguish between PGN patients and controls. (**A**) ROC curve analysis for CD4+/CD8+/TLR2+ T lymphocyte and CD19+/TLR2+ B lymphocyte frequencies (%) in PGN patients; (**B**) sensitivity/specificity curve for CD4+/TLR2+T lymphocytes (%) in PGN patients; (**C**) sensitivity/specificity curve for CD8+/TLR2+T lymphocytes (%) in PGN patients; (**D**) sensitivity/specificity curve for CD19+/TLR2+B lymphocytes (%) in PGN patients.

**Figure 4:**
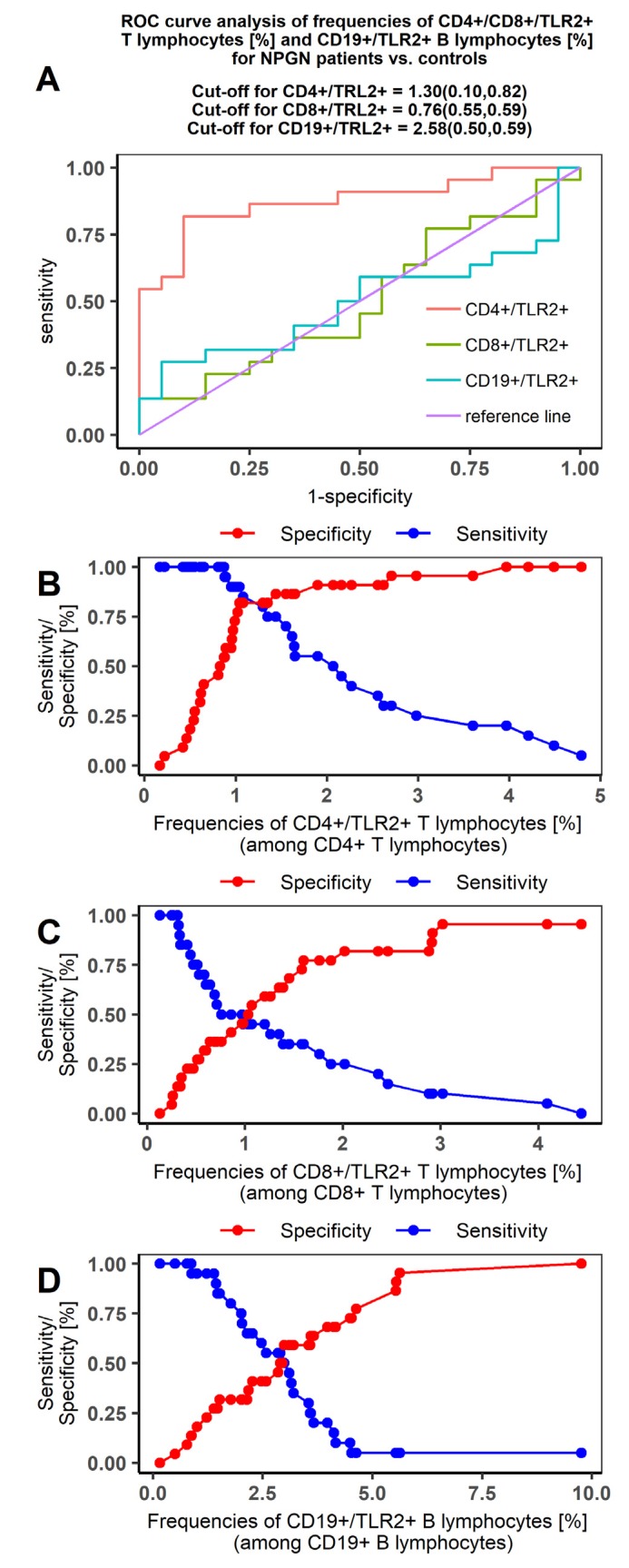
Receiver operating characteristic (ROC) curve analysis on the sensitivity and specificity of TLR2 expression to distinguish between NPGN patients and controls. (**A**) ROC curve analysis for CD4+/CD8+/TLR2+ T lymphocyte and CD19+/TLR2+ B lymphocyte frequencies (%) in NPGN patients; (**B**) sensitivity/specificity curve for CD4+/TLR2+T lymphocytes (%) in NPGN patients; (**C**) sensitivity/specificity curve for CD8+/TLR2+T lymphocytes (%) in NPGN patients; (**D**) sensitivity/specificity curve for CD19+/TLR2+B lymphocytes (%) in NPGN patients.

Our ROC curves comparing the PGN and NPGN groups with controls were characterized by high sensitivity and specificity in the PGN group (Figure 3 and Figure 4, respectively). The AUC values indicated that TLR2 expression was highly specific for distinguishing PGN patients from controls. The most differentiating indicator was TLR2 expression on CD4+ T lymphocytes in PGN patients (AUC = 1; Table 6).

## 3. Discussion

In this work, we investigated the expression of TLR2 on T and B lymphocyte subsets in patients with NPGN and PGN, as well as its relationship with selected clinical parameters. We found that patients had a higher urea and BUN concentration, a lower serum IgG concentration, a lower concentration of serum protein and serum albumin, and a higher quantity of protein after a 24-h urine collection compared to controls. These findings are consistent with the clinical picture associated with glomerulonephritis. Proteinuria, and particularly albuminuria, are characteristic in both PGN and NPGN, leading to a decreased concentration of albumin in serum [21]. As with albumin, immunoglobulins are also passed in the urine in patients with glomerulonephritis, with the accompanying decrease in serum concentration. In nephrotic syndrome, IgG synthesis may be reduced, and its catabolism increased, contributing to the lower serum antibody concentration [22]. Hemoglobin was also significantly lower in PGN patients. This is consistent with the passing of blood in the urine, which is typical of the nephritic syndrome that usually accompanies PGN diseases [5,6].

We have also demonstrated an increased expression of TLR2 on selected subsets of T and B lymphocytes in patients with PGN and NPGN compared to healthy volunteers. The expression of TLR2 on CD4+ T cells, CD8+ T cells, and CD19+ B cells was higher in the PGN group than in the NPGN group and controls. Although TLR2 expression in the NPGN group tended to be higher than in the controls, the difference was significant for the CD4+TLR-2+ subset only. 

Our results suggest that TLR2 plays a role in glomerulonephritis, and particularly in PGN diseases. This is consistent with other findings reported in the literature. Brown et al. demonstrated that TLR2 ligands can activate TLR2 signaling by acting directly on TLR-expressing renal cells, as well as on cells of the innate immune system, such as TLR-expressing T and B cells, to exacerbate glomerulonephritis [20]. Patients with systemic lupus erythematosus, an autoimmune disease associated with both PGN and NPGN, had increased expression of TLR2 mRNA in the peripheral blood mononuclear cells compared to healthy controls [23]. Furthermore, stimulating these cells with ligands for TLR2, TLR4, and TLR9 resulted in the dysregulation of IL-10, TNF-α, and interferon-γ [24]. Liu et al. found that the expression of TLR2 on CD4+ and CD8+ T cells, CD19+ B cells, and CD14+ monocytes was increased in patients with systemic lupus erythematosus [16]. In addition, the in vitro stimulation of TLR2 on CD4+ T cells from patients with systemic lupus erythematosus increased cytokine production. TLR2 has also been implicated in other renal diseases. Tadema et al. found increased expression of this receptor in the peripheral blood monocytes of patients with anti-neutrophil cytoplasmic antibody (ANCA)-associated vasculitis, and also increased proportions of natural killer (NK) cells expressing TLR2, TLR4, and TLR9 [25]. Saito et al. found that TLR2, 3, 5, 7, and 9 mRNAs were upregulated in the peripheral blood mononuclear cells of patients with IgA nephropathy and IgA nephropathy vasculitis with nephritis compared with patients with thin basement membrane nephropathy [18]. Furthermore, the expression of TLR2, 3, 5, and 9 correlated with proteinuria levels in patients with IgA nephropathy. Brown, Sacks, and Robson demonstrated that TLR2 agonists increased the severity of nephrotoxic nephritis in mice through a TLR2-dependent mechanism [26].

We found statistically significant correlations between TLR2 expression and all the selected clinical parameters. The strongest correlations were found in PGN between the frequency of CD4+TLR-2+ cells and urine protein excretion (positive) and the serum concentrations of albumin and IgG (negative). Moreover, the frequency of CD19+-TLR-2+ cells correlated negatively with the IgG concentration. It seems that TLR2 expression on these lymphocyte subsets is a good marker for disease. 

Proteinuria has been associated with TLR2 activation in the kidneys, leading to inflammation in albumin-overloaded nephropathy rats and patients with non-IgA mesangioproliferative glomerulonephritis [27]. In patients with systemic lupus erythematosus and concurrent active nephritis, the level of TLR2 in peripheral blood T cells and of TLR4 in B cells and monocytes was increased, and the rate of protein excretion in the urine was associated with TLR expression in these cells [15].

Our results also show that the expression of TLR2 on these lymphocyte subsets as measured by flow cytometry, particularly on CD4+ T lymphocytes, is a good marker to differentiate PGN from NPGN and healthy controls. While many studies have presented TLR expression in renal tissue as a marker of disease, which would require analyzing a tissue biopsy, the method used in this study is much less invasive and time-consuming. 

Although our observation that TLR-2 expression on immune cells could differentiate between PGN and NPGN seems promising, it needs further validation. In particular, repeated within-subject analyses should examine whether the results remain stable over time. Moreover, the method should be assessed in patients with immune diseases or those receiving immunomodulatory treatment, because such patients are frequently among those with glomerulonephritis.

Further research should be carried out to elucidate the mechanism by which TLR2 activation influences PGN and NPGN. In this study, we grouped patients into the broad categories of proliferative and non-proliferative glomerulonephritis, which encompass many different diseases. Although we showed that TLR2 is expressed more, particularly in patients with PGN, the mechanisms involved may differ according to the etiopathology of each disease. 

## 4. Materials and Methods

### 4.1. Peripheral Blood Collection

Peripheral blood was collected from the ulnar vein of previously untreated patients newly diagnosed with PGN or NPGN and healthy volunteers using sterile, EDTA-coated blood collection tubes (S-Monovette, SARSTEDT, Aktiengesellschaft and Co., Numbrecht, Germany). The diagnosis of glomerulonephritis was made by histological analysis of renal biopsy samples according to standard criteria [28]. The histological analysis included standard hematoxylin-eosin staining and immunohistochemical staining for immune complexes. PGN and NPG were differentiated based on the presence or absence of proliferative changes in the glomeruli [28]. PGN included mesangioproliferative glomerulonephritides (e.g., IgA nephropathy, IgM nephropathy), membranoproliferative glomerulonephritis, and crescentic glomerulonephritis. NPGN included focal segmental glomerulosclerosis, membranous glomerulonephritis, minimal-change disease, and thin basement membrane disease [28]. Fifteen male patients were diagnosed with PGN—eight patients with IgA nephropathy and seven with membranoproliferative glomerulonephritis. Twenty-two male patients were diagnosed with NPGN—11 patients with minimal change disease and 11 patients with membranous glomerulonephritis. The control group comprised 20 male age-matched healthy subjects. Solely male individuals were chosen as a study and control group to avoid the impact of female hormonal fluctuations. Neither the patients nor the controls used immunomodulating agents or hormonal preparations, showed signs of infection within at least three months prior to the study, had undergone blood transfusion, or presented with an autoimmune condition or allergy. Moreover, none of the patients and controls had a history of oncological therapy or prior treatment for tuberculosis or other chronic conditions that could be associated with impaired cellular or humoral immunity. 

The blood samples were then transported to the laboratory for analysis. The diagnosis of glomerulonephritis was in accordance with previously published methodology (Floege and Amman 2016). The study was approved by the Ethics Committee of the Medical University of Lublin (Decision No. KE-0254/290/2014). All patients and volunteers signed an informed consent form before blood collection. 

### 4.2. Flow Cytometry and Sample Preparation

**Figure A1:**
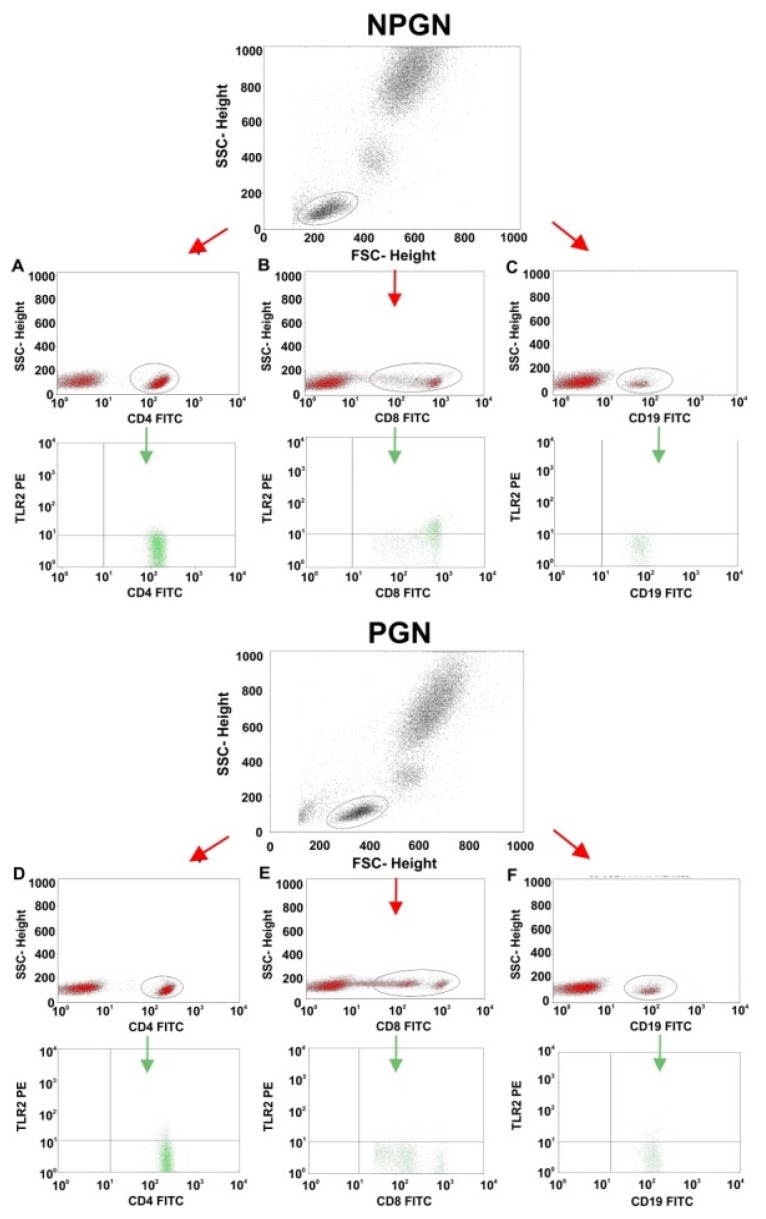
Sample analysis of CD4+/CD8+/TLR2+ T lymphocytes and CD19+/TLR2+ B lymphocytes in NPGN and PGN patients. (**A**). Sample analysis of CD4+/ TLR2+ T lymphocytes in NPGN patients. (**B**). Sample analysis of CD8+/ TLR2+ T lymphocytes in NPGN patients. (**C**). Sample analysis of CD19+/ TLR2+ B lymphocytes in NPGN patients. (**D**). Sample analysis of CD4+/ TLR2+ T lymphocytes in PGN patients. (**E**). Sample analysis of CD8+/ TLR2+ T lymphocytes in PGN patients. (**F**). Sample analysis of CD19+/ TLR2+ B lymphocytes in PGN patients.

**Figure A2:**
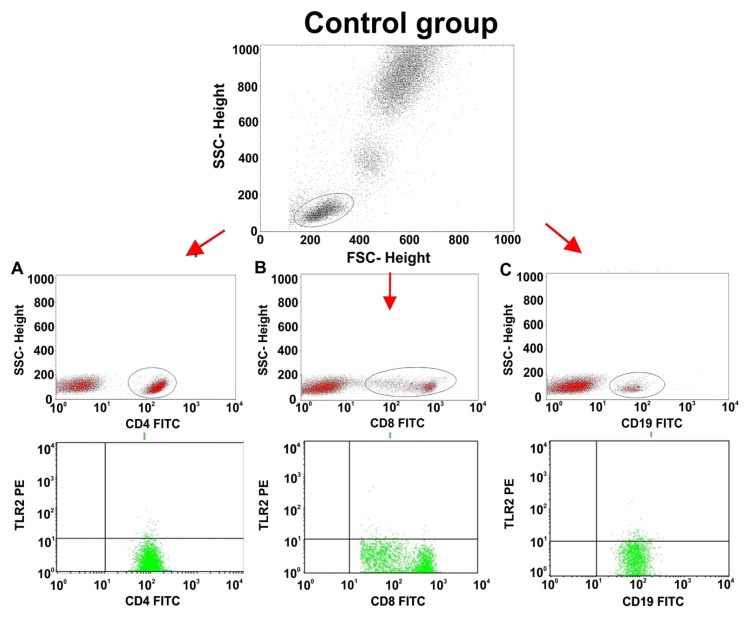
Sample analysis of CD4+/CD8+/TLR2+ on T lymphocytes and CD19+/TLR2+ on B lymphocytes in control patients (**A**–**C**).

**Figure A3:**
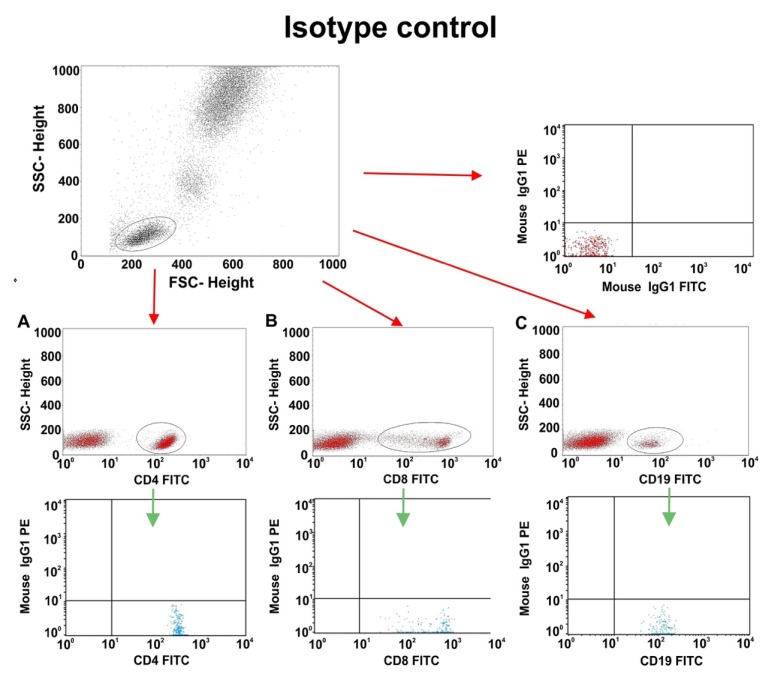
Sample analysis for isotype control CD4+, CD8+, and CD19+ receptors (**A**–**C**).

Flow cytometry was used to determine the percentage of peripheral blood mononuclear cells expressing TLR2. To prepare the material for analysis, 50 µL samples of whole blood from each participant were incubated for 20 min in the dark with pairs of fluorochrome-conjugated monoclonal antibodies against the following markers: CD45 FITC / CD14 PE, CD3 PE / CD19 FITC, CD3 PE / CD4 FITC, CD3 PE / CD8 FITC, TLR 2 PE / CD4FITC, TLR2PE / CD8FITC, and TLR2PE / CD19FITC (BD Biosciences, San Jose, CA, USA). Next, the samples were treated with lysis buffer (Lysing Buffer, BD Pharm Lyse San Jose, CA, USA) then washed in PBS solution (Sigma-Aldrich, Saint Louis, MO, USA). The samples were evaluated using a FACSCalibur flow cytometer (Becton-Dickinson, Franklin Lakes, NJ, USA) equipped with a 488-nm argon laser. For each sample, a minimum of 10,000 events were collected and analyzed using the CellQuest program. The collected data were evaluated using spot plots. A sample analysis for patients with PGN and NPGN is shown in Appendix A Figure A1. Additionally, we measured the percentages of T helper, T cytotoxic, and B lymphocytes. T helper lymphocytes were defined as CD3+CD4+ cells, T cytotoxic lymphocytes were defined as CD3+CD8+ cells, and B cells were defined as CD3-CD19+ cells. The target cell population was determined by using the forward and lateral dispersions (single-color dispersion vs lateral dispersion) and a two-color fluorescence plot. The relative percentage of cells expressing the surface markers was quantified by placing gates around the individual populations. The results are presented as the percentage of CD45+ cells. The percentage of cells was calculated by comparison with the control. Isotype-matched, directly conjugated murine FITC IgG1 κ isotype control and monoclonal antibody control PE IgG1 κ control isotype were used to determine the background signal to exclude contamination and cell aggregates (Appendix A, Figure A2 and Figure A3).

### 4.3. Statistical Analysis

Basic statistics were performed to obtain the mean, median, and standard deviation of each set of data. The Shapiro–Wilk test was used to evaluate the normality of the data distribution. Differences between the groups were analyzed with a Kruskal–Wallis test followed by a Dunn post hoc test; the p-values for the Dunn test were corrected for multiple comparisons with the Bonferroni method. Spearman correlation coefficients were used to study the associations between pairs of variables. Additionally, the diagnostic effectiveness of the laboratory test was determined using ROC curves for parameters related to the patients. The DeLong test was used to compare the respective areas under the curve (AUC) with no effect (AUC = 0.500). The statistical analysis was performed with Tibco Statistica 13.3 (Palo Alto, CA, USA) and Microsoft Excel, and the charts were graphically processed with CorelDraw Home and Student X8 (Ottawa, Canada). Statistical significance was considered when *p* ˂ 0.05.

## 5. Conclusions

In summary, we have shown that the expression of TLR2 is increased on selected lymphocyte subsets in patients with glomerulonephritis, and particularly PGN. Especially on CD4+ T cells, TLR2 expression could be used as a marker to differentiate PGN from NPGN and normal cases. Its use as a non-invasive marker of disease should be further investigated.

## Figures and Tables

**Table 1:** Levels of selected proteins, complement components, and renal function parameters in patients with proliferative (PGN) and non-proliferative (NPGN) glomerulonephritis and controls.

Parameters	NPGN		PGN		Control					
	Mean ± SD	Median (Range)	Mean ± SD	Median (Range)	Mean ± SD	Median (Range)	*p*-Value *	*p*-Value ** (NPGN vs Control)	*p*-Value ** (PGN vs Control)	*p*-Value ** (NPGN vs PGN)
**Age** (years)	41.77 ± 17.61	39.5 (19.00–75.00)	46.00 ± 12.97	43.00 (28.00–70.00)	44.40 ± 12.22	45.00 (20.00–61.00)	0.676 -	0.709	1.000	0.653
**Urea** (mg/dL) (normal range: 15–46)	55.18 ± 31.53	45.12 (17.79–115.66)	35.36 ± 12.62	34.54 (13.03–54.76)	31.40 ± 6.95	32.00 (18.00–42.00)	0.065	0.034	0.781	0.220
**BUN** (mg/dL) (normal range: 7–18)	25.78 ± 14.73	21.08 (8.31–54.05)	16.52 ± 5.89	16.14 (6.09–25.59)	14.67 ± 3.25	14.95 (8.41–19.63)	0.065	0.034	0.781	0.220
**Serum creatinine** (mg/dL) (normal range: 0.7–1.2)	1.15 ± 0.61	0.9 (0.37–2.30)	1.02 ± 0.35	0.95 (0.54–1.79)	0.92 ± 0.12	0.925 (0.70–1.13)	0.962	1.000	1.000	1.000
**Glomerular filtration rate**	85.44 ± 26.87	85.82 (46.80–114.98)	57.19 ± 23.19	56.54 (26.71–100.99)	125.58 ± 10.26	121.08 (114.95–148.19)	<0.001	<0.001	<0.001	0.047
**Serum uric acid** (mg/dL) (normal range: 3.6–8.2)	6.58 ± 1.93	6.35 (3.80–11.90)	6.93 ± 1.53	7.60 (4.00–8.60)	6.22 ± 1.40	6.95 (3.70–7.90)	0.244	0.798	0.143	0.390
**Serum IgG** (g/L) (normal range: 7–16)	4.59 ± 2.47	4.56 (2.00–13.25)	6.96±2.10	6.56 (3.42–10.84)	12.71 ± 1.40	12.79 (10.06–15.47)	<0.001	<0.001	<0.001	0.068
**Serum IgM** (g/L) (normal range: 0.4–2.3)	1.43 ± 0.87	1.05 (0.40–3.00)	1.33 ± 0.74	1.20 (0.50–3.20)	1.66 ± 0.31	1.61 (1.17–2.19)	0.066	0.068	0.067	1.000
**Serum IgA** (g/L) (normal range: 0.7–4)	2.03 ± 0.96	2.07 (0.60–3.64)	3.06 ± 1.44	3.10 (0.77–5.69)	2.39 ± 0.84	2.56 (0.92–3.92)	0.058	0.431	0.252	0.025
**Serum total protein** (g/dL) (normal range: 6.3–8.3)	4.91 ± 1.04	4.90 (3.20–7.40)	5.51±0.92	5.60 (4.20–7.10)	7.35 ± 0.58	7.35 (6.40–8.20)	<0.001	<0.001	<0.001	0.314
**Serum albumin** (g/L) (normal range: 3.40–4.80)	2.53 ± 0.78	2.55 (0.80–3.80)	2.82 ± 0.94	2.96 (0.60–3.90)	4.18 ± 0.36	4.23 (3.50-4.75)	<0.0001	<0.001	<0.001	0.528
**Total protein in a 24 h urine collection test** (g/24 h) (max: 0.15)	5.90 ± 5.31	4.45 (1.00–20.00)	3.85 ± 2.84	3.21 (0.40–9.28)	0.00 ± 0.00	0.00 (0.00–0.00)	<0.0001 (2.86)	<0.001	<0.001	0.0725
**Serum complement component C3** (g/L) (normal range: 0.9–1.8)	1.37 ± 0.29	1.30 (0.90–2.00)	1.19 ± 0.29	1.20 (0.40–1.70)	1.28 ± 0.22	1.24 (0.95–1.78)	0.361	0.475	1.000	0.264
**Serum complement component C4** (g/L) (normal range: 0.1–0.4)	0.32 ± 0.10	0.28 (0.20–0.55)	0.28 ± 0.07	0.27 (0.11–0.46)	0.28 ± 0.08	0.29 (0.15–0.39)	0.663	0.709	1.000	0.625

* Kruskal–Wallis test; ** Dunn test with Bonferroni correction; BUN: blood urea nitrogen.

**Table 2:** Complete blood count and basic lymphocyte subsets in patients with proliferative (PGN) and non-proliferative (NPGN) glomerulonephritis and controls.

Parameters	NPGN		PGN		Control					
	Mean ± SD	Median (Range)	Mean ± SD	Median (Range)	Mean ± SD	Median (Range)	*p*-Value *	*p*-Value ** (NPGN vs Control)	*p*-Value ** (PGN vs Control)	*p*-Value ** (NPGN vs PGN)
**WBC** (10^3^/mm^3^) (normal range: 4.0–10.0)	6.57 ± 1.63	6.4 (4.38–9.80)	7.23 ± 1.85	7.20 (4.30–9.94)	6.82 ± 0.42	6.72 (6.26–7.61)	0.251	0.339	0.998	0.179
**LYM** (10^3^/mm^3^) (normal range: 1.12–4.73)	2.01 ± 0.68	1.96 (1.20–3.70)	2.15 ± 0.79	1.96 (1.20–3.74)	2.50 ± 0.56	2.54 (1.53–3.70)	0.053	0.030	0.136	1.000
**RBC** (10^6^/mm^3^) (normal range: 4.0–5.8)	4.72 ± 1.64	4.40 (3.66–11.80)	4.34 ± 0.50	4.36 (3.34–5.10)	5.17 ± 0.43	5.12 (4.50–5.80)	<0.001	<0.001	<0.001	1.000
**HGB** (g/dL) (normal range: 12–16)	13.33 ± 1.60	13.33 (10.70–16.40)	12.93 ± 1.54	13.10 (9.30–15.10)	14.30 ± 1.19	14.35 (12.50–16.90)	0.030	0.061	0.022	0.816
**PLT** (10^3^/mm^3^) (normal range: 140–400)	244.05 ± 59.02	241.5 (147.00–361.00)	242.40 ± 69.83	214.00 (177.00–410.00)	279.00 ± 57.05	281.50 (186.00–403.00)	0.084	0.110	0.066	1.00
**CD3+ T lymphocytes** (%)	72.26 ± 16.77	76.88 (5.210–87.980)	74.24 ± 6.02	74.71 (63.30–85.07)	72.36 ± 1.76	72.38 (69.99–74.75)	0.175	0.114	0.265	1.000
**CD19+ B lymphocytes** (%)	12.32 ± 13.90	9.52 (1.66–70.30)	11.87 ± 3.80	11.63 (6.97–19.12)	10.60 ± 2.00	10.80 (6.04–14.52)	0.302	0.690	0.596	0.183
**CD3+/CD4+ lymphocytes** (%)	44.04 ± 9.72	44.70 (26.13–63.35)	42.68 ± 5.96	42.84 (34.50–53.26)	42.00 ± 1.21	41.51 (40.54–44.19)	0.300	0.185	0.853	0.602
**CD3+/CD8+ lymphocytes** (%)	28.19 ± 6.71	27.35 (18.44–48.68)	28.10 ± 7.81	25.47 (18.13–42.06)	30.17 ± 1.09	29.97 (28.90–33.17)	0.048	0.047	0.057	1.000
**T CD3+/CD4+:T CD3+/CD8+** lymphocyte ratio	1.69 ± 0.68	1.62 (0.73–3.23)	1.65 ± 0.58	1.68 (0.82–2.74)	1.39 ± 0.06	1.40 (1.24–1.47)	0.136	0.094	0.193	1.000

* Kruskal–Wallis test; ** Dunn test with Bonferroni correction; WBC: white blood cell count; LYM: lymphocytes; RBC: red blood cell count; HGB: hemoglobin; PLT: platelet count.

**Table 3:** Frequencies of TLR2 lymphocytes in patients with proliferative (PGN) and non-proliferative (NPGN) glomerulonephritis and controls.

Parameters	NPGN		PGN		Control					
	Mean ± SD	Median (Range)	Mean ± SD	Median (Range)	Mean ± SD	Median (Range)	*p*-Value *	*p*-Value ** (NPGN vs Control)	*p*-Value ** (PGN vs Control)	*p*-Value ** (NPGN vs PGN)
**CD4+/TLR2+ T lymphocytes** (%)	2.29 ± 1.27	2.12 (0.50–4.79)	16.98 ± 11.18	13.30 (5.12–39.05)	0.82 ± 0.42	0.85 (0.17–1.90)	<0.001	0.003	0.001	0.001
**CD8+/TLR2+ T lymphocytes** (%)	1.35 ± 1.21	0.92 (0.13–4.44)	20.30 ± 15.39	18.43 (2.35–56.60)	1.21 ± 0.90	1.09 (0.25–2.92)	<0.001	1.000	<0.001	<0.001
**CD19+/TLR2+ B lymphocytes** (%)	2.99 ± 2.20	2.74 (0.50–9.76)	19.23 ± 17.93	13.91 (3.82–72.00)	2.71 ± 1.24	2.66 (0.16–5.53)	<0.001	1.000	<0.001	<0.001

* Kruskal–Wallis test; ** Dunn test with Bonferroni correction; TLR2: toll-like receptor 2.

**Table 4:** Correlations between the frequency of TLR-2-positive lymphocyte subpopulations and selected laboratory variables in patients with proliferative glomerulonephritis (PGN) and non-proliferative glomerulonephritis (NPGN).

	CD4 + TLR2 + cells (%)		CD8 + TLR2 + cells (%)		CD19 + TLR2+ cells (%)	
	NPGN	PGN	NPGN	PGN	NPGN	PGN
**Serum urea (mg/dL)**	−0.08, *p* = 0.717	0.05 *p* = 0.863	−0.25, *p* = 0.258	−0.15 *p* = 0.584	−0.12, *p* = 0.589	−0.17 *p* = 0.549
**BUN (mg/dL)**	−0.08, 0.717	0.05 0.863	−0.25, 0.258	−0.15 0.584	−0.12, 0.589	−0.17 0.549
**IgG (g/L)**	−0.01, *p* = 0.980	−0.58 *p* = 0.026	−0.28, *p* = 0.205	−0.21 *p* = 0.442	−0.23, *p* = 0.314	−0.56 *p* = 0.032
**IgA (g/L)**	−0.26 *p* = 0.239	−0.20 *p* = 0.482	−0.03 *p* = 0.912	0.09 *p* = 0.743	−0.13 *p* = 0.554	−0.06 *p* = 0.822
**Total protein (g/dL)**	0.02 *p* = 0.918	−0.43 *p* = 0.111	−0.14 *p* = 0.548	−0.03 *p* = 0.904	−0.22 *p* = 0.319	−0.36 *p* = 0.194
**Albumin (g/L)**	0.09 *p* = 0.674	−0.63 *p* = 0.012	−0.11 *p* = 0.634	−0.34 *p* = 0.218	−0.15 *p* = 0.517	−0.39 *p* = 0.157
**Total protein in a 24 h urine collection test (g/24 h)**	−0.20 *p* = 0.364	0.64 *p* = 0.013	−0.09 *p* = 0.696	0.17 *p* = 0.549	0.16 *p* = 0.465	0.37 *p* = 0.173

Spearman correlation coefficients are shown in upper rows of each cell; significant correlations are marked in bold. BUN: blood urea nitrogen; TLR2: toll-like receptor 2.

**Table 5:** ROC curve analysis to determine the diagnostic accuracy of TLR2 expression on T and B lymphocytes to discriminate between PGN and NPGN patients.

Variable	Frequencies of CD4+/TLR2+ T lymphocytes (%)	Frequencies of CD8+/TLR2+ T lymphocytes (%)	Frequencies of CD19+/TLR2+ B lymphocytes (%)
AUC	1	0.973	0.945
SE (AUC)	0	0.031	0.043
−95%CI	1	0.932	0.880
+95%CI	1	1	1
*Z* statistic	Inf	15.397	10.317
*p* value *	<0.00001	<0.00001	<0.00001

* DeLong test for comparison with no effect (AUC = 0.500). ROC: receiver operating characteristic; AUC: area under the curve; CI: confidence interval; NPGN: non-proliferative glomerulonephritis; PGN: proliferative glomerulonephritis; *p* < 0.05 indicates statistical significance.

**Table 6:** ROC curve analysis to determine the diagnostic accuracy of TLR2 expression on T and B lymphocyte subsets to discriminate between patients and controls.

Variable	Frequencies of CD4+/TLR2+ T lymphocytes (%)		Frequencies of CD8+/TLR2+ T lymphocytes (%)		Frequencies of CD19+/TLR2+ B lymphocytes (%)	
	NPGN vs controls	PGN vs controls	NPGN vs controls	PGN vs controls	NPGN vs controls	PGN vs controls
AUC	0.875	1.000	0.507	0.990	0.498	0.985
SE (AUC)	0.567	0.000	0.903	0.016	0.090	0.020
−95%CI	0.765	1.000	0.313	0.968	0.314	0.956
+95%CI	0.985	1.000	0.674	1.000	0.681	1.000
*Z* statistic	6.619	Inf	0.076	29.873	−0.025	24.093
*p* value *	<0.00001	<0.00001	0.4699	<0.00001	0.4899	<0.00001

* DeLong test for comparison with no effect (AUC = 0.500). ROC: receiver operating characteristic; AUC: area under the curve; CI: confidence interval; NPGN: non-proliferative glomerulonephritis; PGN: proliferative glomerulonephritis; *p* < 0.05 indicates statistical significance.

## Abbreviations

Ig	immunoglobulin
NPGN	non-proliferative glomerulonephritis
PGN	proliferative glomerulonephritis
ROC	receiver operating characteristic
TLR	Toll-like receptor
